# High APACHE II score and long length of bowel resection impair the outcomes in patients with necrotic bowel induced hepatic portal venous gas

**DOI:** 10.1186/1471-230X-11-18

**Published:** 2011-03-09

**Authors:** Jin-Ming Wu, Ming-Shian Tsai, Ming-Tsan Lin, Yu-Wen Tien, Tzu-Hsin Lin

**Affiliations:** 1 Division of General Surgery, Department of Surgery, National Taiwan University, Hospital, Taipei, Taiwan; 2 Division of General Surgery, Department of Surgery, E-Da Hospital, Kaohsiung, Taiwan; 3 Department of Traumatology, National Taiwan University Hospital, Taipei, Taiwan

## Abstract

**Background:**

Hepatic portal venous gas (HPVG) is a rare but potentially lethal condition, especially when it results from intestinal ischemia. Since the literatures regarding the prognostic factors of HPVG are still scarce, we aimed to investigate the risk factor of perioperative mortality in this study.

**Methods:**

We analyzed data for patients with intestinal ischemia induced HPVG by chart review in our hospital between 2000 and 2007. Factors associated with perioperative mortality were specifically analyzed.

**Results:**

There were 22 consecutive patients receiving definite bowel resection. 13 cases (59.1%) died after surgical intervention. When analyzing the mortality in patients after bowel resections, high Acute Physiology And Chronic health Evaluation (APACHE) II score (*p < 0.01*) and longer length of bowel resection (*p *= 0.047) were significantly associated with mortality in univariate analyses. The complication rate was 66.7% in alive patients after definite bowel resection.

**Conclusions:**

Bowel resection was the only potential life-saving therapy for patients with mesenteric ischemia induced HPVG. High APACHE II score and severity of underlying necrotic bowel determined the results in patients after bowel resection.

## Backgrounds

Hepatic portal venous gas (HPVG) was initially reported in neonates with necrotizing enterocolitis in 1955 [1], and subsequently in adults with small bowel infarction [2]. HPVG has been associated with necrotic bowel, Crohn's disease[3], diverticulitis [4], small bowel obstruction [5], and perforating gastric ulcer [5], as well as with iatrogenic conditions such as colonoscopic procedures or barium enema [6,7]. However, the pathogenesis of HPVG is not well understood. In patients with intestinal necrosis induced HPVG, the outcome is usually grave, with mortality being higher than 75% [8]. Until now, the single report that investigated the necrotic bowel inducing hepatic portal venous gas contained less than ten patients [9,10]

## Methods

Between January 2000 and December 2007, 22 consecutive patients that presented on computed tomography (CT) scans with HPVG caused by small- or large-bowel ischemia and received definite bowel resection, were retrospectively reviewed. The diagnosis of HPVG was based on the CT images, which have been interpreted by experienced radiologists. The HPVG was considered associated with bowel ischemia when the patients had clinical presentation of septic shock and peritoneal sign. Furthermore, the CT scans with intravenous contrast enhancement revealed some poorly enhanced bowel loops and/or pneumatosis intestinalis, supporting the diagnosis of intestinal necrosis. Six cases received medical treatment only because of profound shock and multiple organ failures. All patients in our study had the pathological diagnosis of necrotic bowels. The demographic data was recorded. Clinical findings were classified as abdominal pain, fever (body temperature >38.0 degrees Celsius), shock, and disease severity (evaluated by the Acute Physiology And Chronic Health Evaluation [APACHE ] II score). Preoperative laboratory findings were recorded. All blood test results were collected from the available last findings prior to surgery. Surgical findings were recorded as: (1) intestinal necrosis involving small bowel only, large bowel only, or both (2) the total length of bowel resection (cm). The study was performed with the approval of the ethics committee of the National Taiwan university Hospital (Reference number: 201101031RC).

### Statistical analysis

Statistical analyses were performed using the SAS statistical software (Version 9.1.3, SAS Institute Inc., Cary, NC, U.S.A.) and the S-PLUS statistical software (Version 6.2.1, Insightful Corporation, Seattle, WA, U.S.A.) The associations between categorical variables were examined by chi-square test or Fisher's exact test, whereas the differences in the means of continuous variables were analyzed by Student's independent two-sample *t *test.

## Results

We did analysis of mortality to the 22 patients who underwent bowel resection. Descriptive analyses of demographic and clinical characteristics and outcomes between the two groups can be compared in Table 1. Among the 22 subjects, there were 11 male (50.0%) and 11 female (50.0%) patients. The mean age was 68.9 years. There were 6 heavy-smokers. There were 11 hypertension, 10 diabetes mellitus, and 8 chronic renal failure patients, respectively. With regards to clinical findings, all patients had abdominal pain, 13 had fever, and 17 had shock, respectively. The mean scores of APACHE II were 11.9 in surviving patients, and 14.3 in non-survivors. The APACHE II score was significantly lower in surviving patients than in non-survivors (*p <*0.01).

**Table 1:** The characteristics, clinical, laboratory, surgical findings, and results

Variable	All patients (n = 22)	Survivors (*n *= 9)	Non-survivors (*n *= 13)	***P*****#**
Male:female ratio	11:11	4:5	7:6	0.999
Age (years) (mean ± SD)	68.9 ± 17.6	71.0 ± 8.9	67.4 ± 21.9	0.646
Smoking history	6	3	3	0.655
Comorbidity:	17	6	11	0.609
Atrial fibrillation	5	2	3	0.999
Coronary arterial disease	5	1	4	0.360
Hypertension Diabetes	11	4	7	0.999
mellitus Chronic renal	10	3	7	0.415
failure	8	4	4	0.662
COPD	6	3	3	0.415
Cancer	3	1	2	0.999
Clinical presentations:				
Fever	13	5	8	0.999
Shock	17	5	12	0.253
APACHE score	18.6 ± 3.8	15.1 ± 2.8	19.9 ± 3.2	<0.01
Abdominal pain	22	9	13	0.999
Image findings				
Pneumatosis intestinalis	19	7	12	0.544
Laboratory findings				
Hb (g/dl)		12.5 ± 2.1	11.3 ± 1.8	0.165
WBC (x10^3^/μl)		13677 ± 11521	14149 ± 6353	0.903
Platelet (x10^3^/μl)		181.9 ± 120.4	144.7 ± 100.8	0.441
PT (sec)		15.3 ± 2.8	18.2 ± 7.6	0.244
aPTT (sec)		39.6 ± 11.4	41.5 ± 13.3	0.745
Glucose (mg/dl)		239.6 ± 94.0	173.8 ± 63.3	0.085
Total bilirubin (mg/dl)		1.1 ± 1.0	2.4 ± 2.0	0.099
Urea (mg/dl)		48.2 ± 28.1	48.9 ± 25.4	0.947
Creatinine (mg/dl)		2.8 ± 1.7	2.7 ± 2.0	0.898
Sodium (mmol/L)		136.9 ± 7.3	133.2 ± 7.8	0.270
Potassium (mmol/L)		4.0 ± 0.5	4.6 ± 1.0	0.110
Arterial pH		7.4 ± 0.1	7.4 ± 0.1	0.477
Bicarbonate (mmol/L)		18.0 ± 5.9	17.6 ± 5.3	0.867
Surgical results				
Morbidity	NA	6	NA	NA
Mortality	13	0	13	

NA: no data available

Data are mean (SD) or number unless otherwise indicated.

COPD = chronic obstructive pulmonary disease.

In addition to HPVG in CT, 19 of the 22 patients had pneumatosis intestinalis. Table 2 describes the surgical findings and results. Regarding the location of necrotic bowels, 21 cases involved the small intestine, 8 involved the large intestine, 7 involved both the large and small bowels, and 1 involved large bowel only. All cases received resection of ischemic bowel and end stoma. There were 14 small bowel resection, 4 small bowel resection plus right hemicolectomy, 3 small bowel resection plus total colectomy, and 1 subtotal colectomy in our study. The mean length of bowel resection was 215.8 cm. The mean length of bowel resection was significantly shorter in survivor cases than in non-survivors (*P *= 0.047).

**Table 2:** Compare the surgical findings and results

Variable	All patients (n = 22)	Survivors **(*****n *****= 9)**	Non-survivors **(*****n *****= 13)**	***P***
*Surgical findings*				
Length of bowel resection (cm)	215.8 ± 123.5	162.7 ± 133.9	284 ± 67.5	0.047
Only small bowel involved	14	7	7	0.380
Both small & large bowels involved	7	1	6	0.165
Only large bowel involved	1	1	0	0.490
*Method of operation*				
Only small bowel resection	14	7	7	
Small bowel resection + right hemicolectomy	4	1	3	
Small bowel resection + total colectomy	3	0	3	
Subtotal colectomy	1	1	0	
*Morbidity*	NA	6	NA	NA
Pneumonia		2		
Deep wound infection		2		
Acute renal failure		2		

There were 13 perioperative deaths (59.1%) among all 22 patients and all died from multi-organ failure and septic shock. 6 postoperative complications (66.7%) were observed in 9 surviving patients. The most common complications were 2 cases of pneumonia, 2 cases of wound infections requiring further debridement, and 2 cases of acute renal failure.

## Discussion

Three mechanisms of HPVG formation have been proposed: (a). mucosal damage and lumenal gas escape to the portal system via portal microvenule, (b). septic embolization or abscess that rupture through the small portal venules, (c). gas forming portal pyemia [8,9]. Other less important factors are increased lumenal pressure such as during colonoscopy or large bowel enema which are associated with HPVG. The first two theories are using principles in Physics, which directs gas passing from breaks of intestinal mucosa into the mesenteric venous system. The third theory focuses on a biological phenomenon of bacteremia causing sepsis, and finally all gas that formed in the mesentery is accumulated in the hepatic portal venous system and becomes a significant finding in image studies.

In our study, high APACHEII score was significantly associated with poor prognosis. One report [11] addressed that APACHE II score was a prognostic factor in patients with ischemic bowel. The other [12] had similar findings in patients who develop acute abdominal complications in a medical intensive care unit. APACHE II score is designed to measure the severity of disease for adult patients admitted to intensive care units. Although patients suffering from necrotic bowels induced HPVG often had poor prognosis, APACHE II score may be a useful prognostic tool for physician to judge the results.

In this study, longer length of bowel resection was associated with poorer prognosis that implied the severity of underlying necrotic bowel. The surgeons may do a second-look operation for borderline ischemic bowel that could preserve extra bowel length in selected patients. According to our experiences, no patient received a second-look operation because the vital signs were very unstable. Surgeons preferred definite resection that may stop profound and proceeding shock. Otherwise, some cases need home total parenteral nutrition. Definite bowel resection was the only life-saving way for theses patients. However, the complication rate in survivors was as high as 66.7%. In the past, HPVG was considered an "ominous finding" if associated with ischemic bowel disease. Recent reports on mortality in ischemic bowel disease with HPVG were described from 55% to 66.7% [13-15]. The reason of decreased mortality rates in these reports may be associated with early detection of CT scanograms and advancement of critical care. However, HPVG may just be a radiological finding when the underlying disease is benign. The patients recover uneventfully.

In our series, the mortality was lower. There may be several reasons. First, we arranged CT scanograms with contrast if acute mesenteric ischemia was suspected, especially in uncooperative patients. In our experience, some patients receiving major operation (cardiopulmonary bypass or craniectomy) could not resume their consciousness during perioperative courses and the abdominal presentation of ischemic bowel may be more subtle. Contrast enhanced CT scans may impair renal function but offer more abdominal information. The sensitivity and specificity rates of multi-detector row CT are 92-96% and 94-100%, respectively [16-18]. Second, it may be associated with early use of total parenteral nutrition (TPN). Patients could not gain the energy through the enteral way after major bowel resection and shock status. As a result, we started TPN support as soon as possible and supplement of albumin when the value was less than 3.0 mg/dl. Third, we used aggressive artificial organ support in selected patients, such as renal replacement therapy and extracorporeal membrane oxygenation.

The present study has some limitations. First, this was a retrospective observation study. Second, there were relatively few cases in our study, despite it is the largest series so far. Most of the reports in the literature of ischemic intestines inducing HPVG were all small-numbered case series [15,19,20]. Third, we tried to analyze if the timing of operation was a significant factor. However, the correct time of actual mesenteric ischemia was difficult to define, especially in unconscious patients. Other laboratory tests such as arterial blood gas and lactate level should be checked especially in unconscious patients to detect the severe sepsis earlier. We agreed that early bowel resection is the most important element to save theses patients. When mesenteric ischemia was suspected, imaging study, such as CT or angiography, should be considered. HPVG was not only a "poor" imaging finding but also a clue of acute abdomen.

## Conclusions

Patients with HPVG detected on CT scan had a poor prognosis if the underlying disease was associated with ischemic bowel disease. Only bowel resection could afford the chance of survival in selected cases. Patients with high scores of APACHE II and longer length of bowel resection are associated with poor prognosis.

## Competing interests

The authors declare that they have no competing interests.

## Authors' contributions

J-M Wu, M-S Tsai, and T-H Lin conceived the idea, drafted the manuscript, and analyzed the data. M-T Lin contributed to data interpretation and revised the manuscript. Y-W Tien contributed to data verification and revised the manuscript. All authors have participated in the data analysis and reporting stage of this manuscript, and have seen and approved the final version.

## Pre-publication history

The pre-publication history for this paper can be accessed here:

http://www.biomedcentral.com/1471-230X/11/18/prepub
